# Preventable Causes of Insanity

**Published:** 1911-12

**Authors:** 


### Preventable Causes of Insanity.

An element of hope appears in modern discussions of insanity like that of Mr. Homer Folks in the May (1911) *Review of Reviews,* since they show that a considerable portion of insanity is entirely preventable by abstinence from alcohol and avoidance of the dangers of syphilis.

Dr. Thos. W. Salmon of the Public Health and Marine Hospital Service pointed out three years ago (*Popular Science Monthly* 1908) that more than one-fourth of all first admissions to the New York hospitals were due to general paralysis and alcoholic insanity, about evenly divided. The influence of city life was indicated by the larger proportion of general paralytics who came from the city—three to one of the men and two to one of the women. But the cases of alcoholic insanity among the women were seven city dwellers to one from the country, showing, in Dr. Salmon's opinion, the influence of the back room and ladies' entrance of the city saloons.

Considering the men separately, forty-two per cent of those from the city suffered either from general paralysis or alcoholic insanity.

Dr. Archibald Reid of England says there is no doubt that syphilis and alcohol are directly responsible for more cases of mental derangement than any other two causes. Referring also to Dr. Salmon's work, Dr. Reid thinks the latter is absolutely correct when he says that the lesson of these statistics is full of hope, for it shows that a large proportion of all cases of insanity come from causes within our own control and are, therefore, preventable.—*Scientific Temperance Federation.*

---

As part of a hernioplasty it is always worth while to reduce by sutures the hiatus in the transversalis fascia whenever this can be conveniently done.—*American Journal of Surgery.*

